# Editorial: Recent advances in thermal and nonthermal ablative technologies of the thyroid

**DOI:** 10.3389/fendo.2025.1601452

**Published:** 2025-04-24

**Authors:** Pia Pace-Asciak, Ralph P. Tufano

**Affiliations:** ^1^ Department of Otolaryngology – Head and Neck Surgery, University of Toronto, Toronto, ON, Canada; ^2^ Head and Neck Endocrine Surgery, Sarasota Memorial Care System, Florida State University School of Medicine, Sarasota, FL, United States

**Keywords:** thyroid disease, radiofrequency ablation, laser ablation, benign and malignant thyroid nodules, ablative therapy

The field of thyroidology is rapidly expanding to include alternatives beyond surgery such as minimally invasive thermal and nonthermal ablative techniques for benign and carefully selected malignant thyroid nodules. The patient-centered, minimally invasive approach to thyroid nodules has been used globally for years and is now permeating North America. The main appeal for these newer approaches is that compared to surgery it avoids the need for a general anesthetic, an incision, and is associated with a quick recovery and low risk for hypothyroidism. We compiled a series of articles that reflect this exciting time in thyroid research with articles that look at different therapeutic and diagnostic approaches for treating benign and malignant thyroid nodules. Within this diverse set of articles, we share quality research that looks at ways to individualize treatment ranging from isolating biomarkers at the microscopic genetic level for autoimmune thyroiditis spanning to novel diagnostic and clinical approaches for treating thyroid nodules with ablative technologies.

With the introduction of any new procedure, there is always a learning curve to achieve proficiency. This is a timely topic given the number of new centres bringing thermal ablation to their institution and wanting safe alternatives for their patients. According to the literature, physicians with prior experience doing their own thyroid ultrasounds and ultrasound guided fine needle biopsy (FNA) may have to carry out at least 20-30 radiofrequency ablation (RFA) procedures before reaching a safe and competent level (1). Chytiris et al., explore this concept further by providing insight into the learning curve for safely and effectively ablating non-functioning thyroid nodules with the nodule volume considered. We felt this paper was important since the authors provide guidance to novice operators on the various factors (such as technical ability and nodule characteristics) involved in successful and safe ablation.

Ultrasound is the gold standard for risk stratifying whether biopsy is warranted. Traditional grey scale ultrasound can be ineffective for managing isoechoic thyroid nodules and ruling out sonographic features of cancer (2). Most of these nodules are benign but can lead to unnecessary biopsies in the process of ruling out malignancy. Goundan et al. discuss the use of quantitative ultrasound (QUS) as a novel imaging method to assess the tissue microarchitecture of the nodule as a means of further risk stratifying isoechoic and hypoechoic thyroid nodules. Improving accuracy of imaging by subtyping the nodule characteristics may lead to less FNA biopsies, and improved quality of care.

We have included articles that describe methods for ensuring safety and efficacy when using Radiofrequency ablation (RFA). By pushing the boundaries and indications of RFA, Teliti et al. ensure patient safety by monitoring the recurrent laryngeal nerve with flexible laryngoscopy in the awake patient while using RFA in bilateral thyroid nodules. As this technology continues to evolve, innovative techniques emerge to ensure efficacy and safety but also accuracy. Sarkis et al. use a novel guide when performing RFA of thyroid nodules to provide a robust in line visualisation of the 7 mm or 10 mm radiofrequency active probe tip to ensure safety while near the danger triangle and complete ablation of the conceptual subunits during RFA to avoid later regrowth. Furthermore, Chuanke et al. demonstrates long-term volume reduction in up to 6 years post treatment, with stabilization 2 years post treatment. Comparing preoperative contrast-enhanced ultrasonography (CEUS) in the target area with postoperative CEUS to ensure complete ablation particularly along the margins of the nodule is a useful technique ensuing long-term success. In addition, the use of percutaneous laser ablation has been shown to effectively reduce nodule volume and alleviate associated compressive symptoms in benign nodules. Malik et al. demonstrate sustained reduction long-term (up to 18 months) post laser ablation with a thin percutaneous optic fiber creating a predictable zone of coagulation with a type of image guidance (spatial overlay) for treatment planning to ensure all zones are equally targeted.

While early studies indicate that thermal ablation does not appear to compromise the overall survival rate or increase the risk of recurrence, more extensive long-term data are required to better understand its role in the management of thyroid cancer. The use of thermal ablation, such as RFA, in the treatment of low-risk papillary thyroid carcinoma (PTC) is an area of growing interest. PTC, particularly in its low-risk form, has a relatively good prognosis, and many patients may not require aggressive surgical intervention or treatment at all. For these individuals, thermal ablation may provide a less invasive option, with the potential to avoid the risks associated with thyroidectomy. Gong et al. compare the efficacy, safety and impact on the quality of life between thermal ablation (RFA and laser) and surgical interventions in patients with papillary microcarcinoma. A superior quality of life and better functional outcomes were noted in the thermal ablation group compared to the surgery group underscoring the need to incorporate such treatments for low-risk disease.

As the use of RFA expands, its application in low-risk papillary thyroid carcinoma offers a potentially transformative approach to treatment, though more long-term data are needed to fully evaluate its role in oncological management. On the other end of the spectrum, newer studies continue to push the boundaries of traditional treatment methods and use creative multimodal solutions for more aggressive thyroid disease. Wan et al. explore the clinical benefits of using ultrasound guided ^125^Iodine seed implantation for iodine-refractory differentiated thyroid cancer (RAIR-DTC). These authors employ a localized treatment to tumor cells which causes minimal damage to surrounding structures by emitting energy over a long period of time compared to doses of external beam radiation which causes lethal damage to surrounding tissues. Akin to the concept of minimally invasive thermal ablation, this paper employs other methods for targeted local tumor ablation with minimal side effects.

Complications can and do happen despite the excellent safety profile of minimally invasive treatments. Major complications are rare with minimally invasive techniques but do exist. Ferraro et al. review the literature and share a case report of thyroid nodule rupture post RFA and propose a treatment algorithm for managing major complications. Chuanke et al. also share a complication of Horner’s syndrome post cervical chain injury for readers so awareness and consent can be appropriately obtained.

At a more microscopic level, the genetics of thyroid disease can dictate the trajectory of disease outcome. With autoimmune thyroiditis, understanding the complex genetic expression of T cell exhaustion (Tex) and its role in Graves’ disease (GD) can be helpful to tailor therapeutics. Jiang et al. examine gene profiling in GD to investigate how Tex related gene CBL is expressed in autoimmune thyroid disease and to subtype its expression. By shedding light on the genetic expression of the immune response within the thyroid, it introduces a novel research avenue for developing molecularly targeted drugs for each subtype. Once again, another example of how the field of thyroidology is moving away from a “one size fits all” approach to a more tailored and individualized one.

We hope you enjoy the diverse topics within our Research Topic on ablative technology and become inspired to develop innovative approaches within your own centres for further improving the management of both benign and malignant thyroid disease.
